# Targeting PAI-1 in Cardiovascular Disease: Structural Insights Into PAI-1 Functionality and Inhibition

**DOI:** 10.3389/fcvm.2020.622473

**Published:** 2020-12-22

**Authors:** Machteld Sillen, Paul J. Declerck

**Affiliations:** Laboratory for Therapeutic and Diagnostic Antibodies, Department of Pharmaceutical and Pharmacological Sciences, KU Leuven, Leuven, Belgium

**Keywords:** plasminogen activator inhibitor 1 (PAI-1), PAI-1 inhibitors, serpin (serine proteinase inhibitor), fibrinolyisis, cardiovascular disease

## Abstract

Plasminogen activator inhibitor-1 (PAI-1), a member of the serine protease inhibitor (serpin) superfamily with antiprotease activity, is the main physiological inhibitor of tissue-type (tPA) and urokinase-type (uPA) plasminogen activators (PAs). Apart from being crucially involved in fibrinolysis and wound healing, PAI-1 plays a pivotal role in various acute and chronic pathophysiological processes, including cardiovascular disease, tissue fibrosis, cancer, and age-related diseases. In the prospect of treating the broad range of PAI-1-related pathologies, many efforts have been devoted to developing PAI-1 inhibitors. The use of these inhibitors, including low molecular weight molecules, peptides, antibodies, and antibody fragments, in various animal disease models has provided ample evidence of their beneficial effect *in vivo* and moved forward some of these inhibitors in clinical trials. However, none of these inhibitors is currently approved for therapeutic use in humans, mainly due to selectivity and toxicity issues. Furthermore, the conformational plasticity of PAI-1, which is unique among serpins, poses a real challenge in the identification and development of PAI-1 inhibitors. This review will provide an overview of the structural insights into PAI-1 functionality and modulation thereof and will highlight diverse approaches to inhibit PAI-1 activity.

## Introduction

Hemostasis is an essential physiological process that preserves the integrity of the vascular system and secures sufficient blood flow throughout the circulatory system. The balance between clot formation (coagulation) and clot dissolution (fibrinolysis) is very tightly regulated in a spatiotemporal manner and requires a dynamic interplay with other systems involved, such as the vascular system and platelets (1). Briefly, upon vascular injury, a sequence of cellular and molecular events is triggered that can be characterized by three distinct but overlapping phases of initiation, amplification, and propagation (coagulation) (2, 3). The end result of the coagulation cascade is the conversion of fibrinogen, a soluble plasma protein, into an insoluble fibrin meshwork that constitutes blood clots. To limit the coagulatory response to the site of injury and prevent vascular occlusion, the prothrombotic response is balanced by the fibrinolytic system. Fibrinolysis revolves around the enzymatic activation of plasminogen into the key fibrinolytic enzyme plasmin through tissue-type (tPA) and urokinase-type (uPA) plasminogen activators (PAs) (4). Tissue-type PA is produced by vascular endothelial cells and released in response to thrombin and venous occlusion. It is primarily involved in the activation of plasminogen that is required for fibrin dissolution in the circulation (5, 6). In contrast, uPA is expressed by a variety of cells, including renal epithelial cells, inflammatory cells, and cancer cells. It is considered more important in pericellular proteolysis during tissue remodeling and cell migration through the activation of cell-bound plasminogen (7, 8). Plasminogen activator inhibitor-1, a member of the serpin superfamily, is a key component of the plasminogen/plasmin system as it is the primary inhibitor of tPA and uPA.

## Synthesis, Distribution, and Biochemical Properties of PAI-1

PAI-1 was first detected almost four decades ago as an inhibitor of the fibrinolytic system associated with cultured bovine endothelial cells (9). Not much later, several research groups demonstrated its presence in human plasma (10–12), as well as various other cell types throughout the body, including the spleen, liver, kidney, lung, and adipocytes, albeit at different concentrations and with variable functional activities (13, 14). Furthermore, PAI-1 expression and release are strongly regulated by various factors, including growth factors (e.g., transforming growth factor-β, epidermal growth factor), inflammatory cytokines (e.g., tumor necrosis factor-α and interleukin-1β), hormones (e.g., insulin, glucocorticoid, and angiotensin II), glucose, and endotoxin of Gram-negative bacteria (15, 16). In the blood, PAI-1 occurs in two distinct pools, free in plasma or retained in platelets (17). Plasma PAI-1 circulates mainly in the active conformation at relatively low levels (5–50 ng/mL) (17) showing a large interpersonal variability caused by factors including race/ethnicity (18), gender (19), and body composition (20). In contrast, platelet PAI-1 serves as the main blood pool of PAI-1 with concentrations up to ~300 ng/mL (17). Initially, several studies showed that platelet-derived PAI-1 is less active compared to plasma PAI-1, considered being only 2–5% functionally active (21, 22). However, the pre-analytical methods used in these studies, such as sonication or freeze-thawing, may have reduced the activity of platelet-derived PAI-1 since more recent studies were able to demonstrate a substantially higher activity for PAI-1 (23, 24). Even though platelets do not contain a nucleus, they retain the ability for *de novo* PAI-1 synthesis through translationally active PAI-1 messenger RNA, of which the synthesis rate is importantly increased by platelet activation (23). As a result, at least 50% of platelet-derived PAI-1 was shown to be in the biologically active form and capable of forming an irreversible PAI-1/tPA complex. Importantly, platelet-derived PAI-1 has a substantial role in conferring thrombolysis resistance to the clot through local accumulation caused by its release from activated platelets and subsequent partial retention of functional PAI-1 on the platelet membrane (24–26).

The 12.3 kb human PAI-1 gene (*SERPINE1*) was mapped to chromosome 7 (7q21.3-q22) and contains nine exons and eight introns (27, 28). The exons encode for a 23 amino acid long signal peptide and the 379 amino acid long mature PAI-1 protein (29). Additionally, a mature form comprising 381 amino acids, including two extra amino-terminal (N-terminal) residues, has been identified and is most likely the result of cleavage at an alternative cleavage site for signal peptidases (30). Native PAI-1 is a 45-kDa single-chain glycoprotein that lacks cysteines. Based on the amino acid sequence, three potential sites for N-linked glycosylation were identified of which Asn209 and Asn165 display a heterogeneous tissue-type specific glycosylation pattern while Asn329 is not utilized *in vivo* (31, 32). Even though glycosylation often has a critical role in determining protein structure, function, and stability for many proteins, glycosylation of PAI-1 is not a prerequisite for its ability to inactivate PAs or to interact with its cofactor vitronectin (33). In contrast, several studies demonstrated that glycosylation can have a tremendous effect on the neutralizing activity of PAI-1 inhibitors and therefore emphasizes the significance of the source of PAI-1 used in the development of PAI-1 inhibitors (31, 34, 35).

## Structural and Functional Properties

### PAI-1 Is an Inhibitory Serpin

The serpin superfamily comprises over 1,500 inhibitory and non-inhibitory proteins that are broadly distributed among several species, including humans, animals, viruses, bacteria, and plants (36). Despite their profound structural similarity, serpins are functionally very diverse. Whereas, their biological function often requires inhibition of proteases, some non-inhibitory serpins function as, for example, hormone transporters (37), tumor repressors (38), or molecular chaperones (39). Based on their evolutionary relatedness, eukaryotic serpins have been divided into 16 clades (termed A-P), with clades A-I representing human serpins. PAI-1 is categorized as a clade E serpin and is considered to be the main physiological inhibitor of tPA and uPA. However, other serpins with inhibitory activity toward PAs have been identified and include plasminogen activator inhibitor-2 (clade B), protease nexin I (clade E), and neuroserpin (clade I) (40).

**Figure 1:**
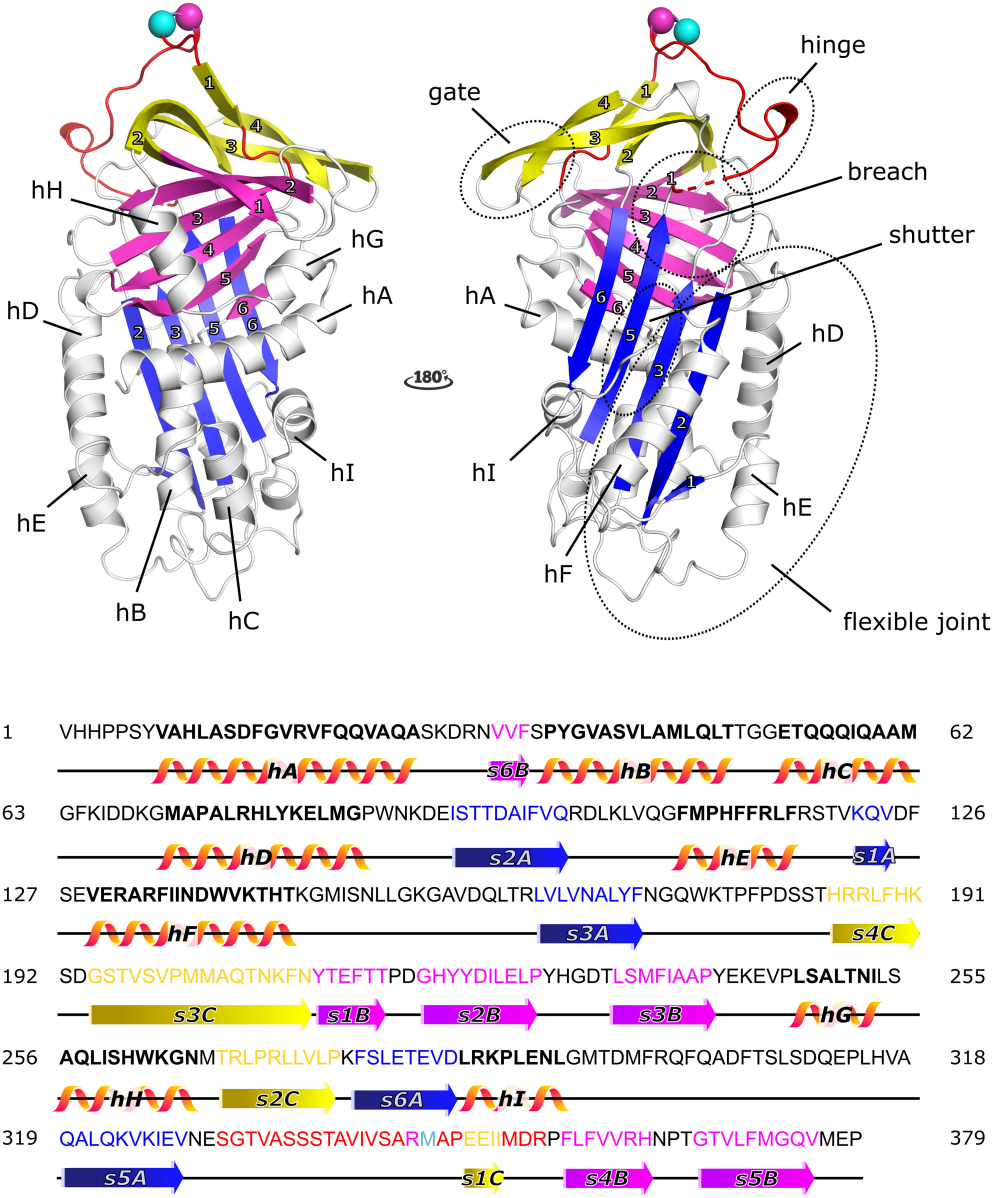
Cartoon representation of the crystal structure of the active form of plasminogen activator inhibitor-1 (PAI-1) [PDB ID 6ZRV (41)] and the amino acid sequence of native PAI-1. PAI-1 shows the evolutionarily conserved topology of serpins, consisting of three β-sheets (A–C) and nine α-helices (hA-hI). β-sheet A, B, and C are shown in blue, magenta, and yellow, respectively, with numbers labeling the individual strands. The α-helices are indicated in the figure. The reactive center loop (RCL) of PAI-1 connects strand 5 of β-sheet A (s5A) to strand 4 of β-sheet B (s4B) and comprises strand 1 of β-sheet C (s1C). The RCL is shown in red, with the reactive center Arg346 (P1) and Met347 (P1′) represented by a magenta and cyan sphere, respectively. Other important domains that control and modulate PAI-1 conformational changes (the gate, hinge, breach, shutter, and flexible joint regions) are also indicated. Residues missing in the crystal structure are indicated by a dashed line. The amino acid sequence is presented and secondary structures (α-helices and β-strands) are indicated in the colors corresponding to the cartoon representation.

PAI-1 displays the well-conserved structure of serpins (Figure 1), characterized by three β-sheets [termed A–C, with strand numbers indicated as s(#)A, s(#)B, and s(#)C] and nine α-helices (termed hA-hI) (42, 43). As the primary inhibitor of PAs, PAI-1 rapidly inactivates both tPA and uPA with second-order rate constants between 10^6^ and 10^7^ M^−1^ s^−1^ following the basic mechanism applied to all serpin/serine proteinase reactions (43, 44). The key to this reaction is that the PA recognizes PAI-1 as a (pseudo)substrate. Therefore, PAI-1 carries a flexible surface-exposed reactive center loop (RCL) of 26 residues long (331-SGTVASSSTAVIVSA*RM*APEEIIMDR-356, designated P16-P10′) that presents a substrate-mimicking peptide sequence (Arg346-Met347, designated as P1-P1′). PAI-1 is synthesized in a metastable active conformation, i.e., with the RCL protruding from the top of the molecule, which is essential for the kinetic trapping of PAs in a thermodynamically favorable complex. Several regions–the hinge region (P15-P9 of the RCL), the breach region (the top of β-sheet A), the shutter domain (the central part of s3A and s5A and the N-terminal part of hB), the gate region (s3C and s4C), and the flexible joint region (hD, hE, hF, and s1A)–have been shown to be important in controlling and modulating PAI-1 functionality through conformational changes (Figure 1).

### Mechanism of Protease Inhibition

**Figure 2:**
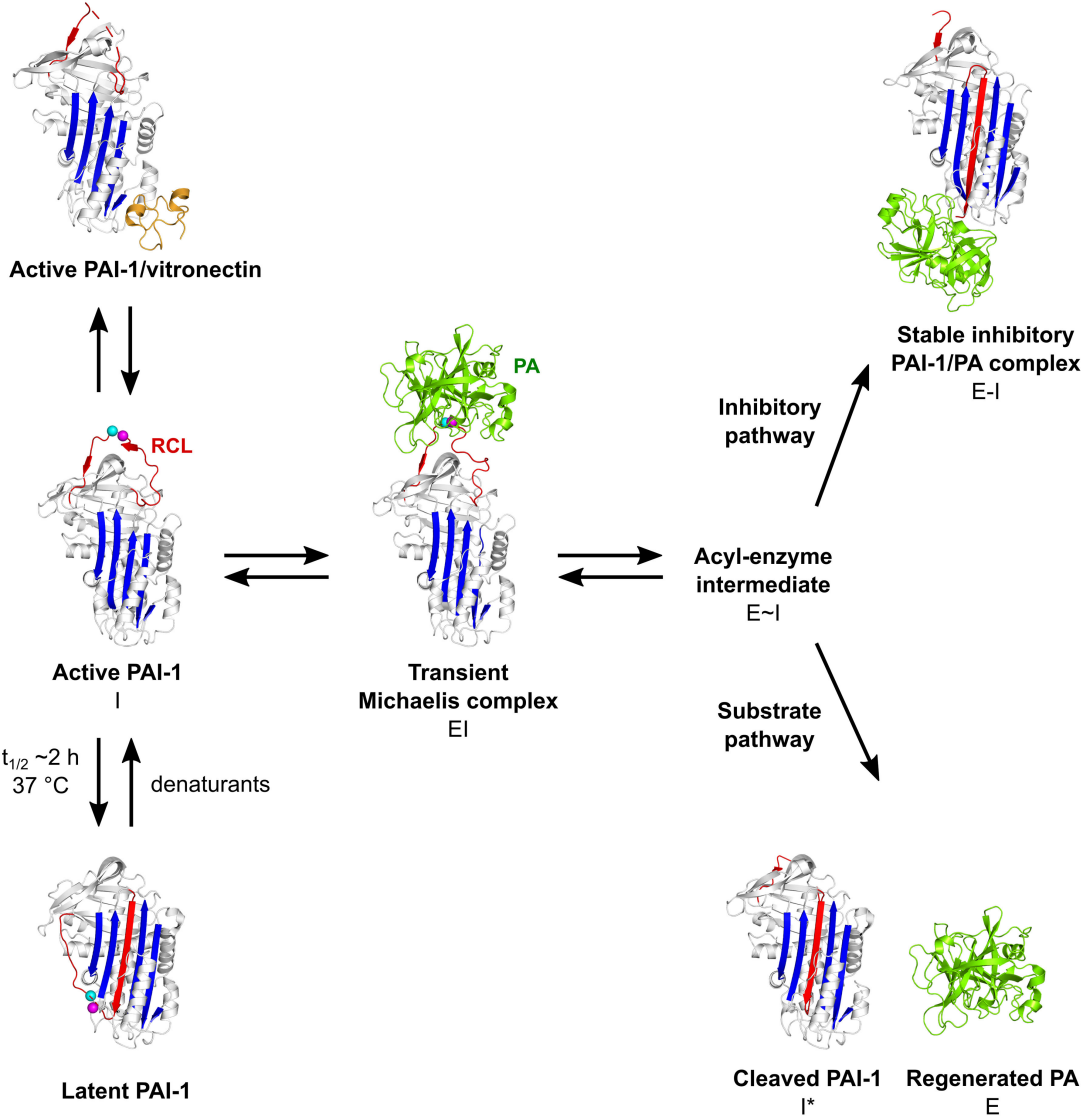
Schematic overview of the PAI-1 (I) conformations as well as its interactions with plasminogen activators (PAs, E) and cofactor vitronectin. Following the formation of a non-covalent PAI-1/PA Michaelis complex (EI), the P1-P1′ bond is cleaved to generate an acyl-enzyme intermediate (E~I). From here on, the reaction proceeds through a branched pathway, resulting in either the formation of an irreversible inhibitory complex (E-I) or the generation of cleaved PAI-1 (I*) due to the hydrolysis of the acyl-enzyme intermediate. PAI-1 is shown in white; the central β-sheet A of the PAI-1 molecule in blue; the flexible reactive center loop (RCL) in red, and Arg346 and Met347 (P1-P1′) of the reactive center are indicated by magenta and cyan spheres, respectively. The PA is shown in green. Vitronectin is shown in orange. PDB structures 1DVN (45), 1DB2 (46), 5BRR (47), 3EOX (48), 1EZX (49), 1H4W (50), and 1OC0 (51) were used to generate this figure. Figure adapted from Sillen et al. (52).

The PAI-1/PA reaction is initiated by the formation of a non-covalent 1:1 stoichiometric Michaelis complex (EI) between PAI-1 (inhibitor, I) and the PA (enzyme, E) (Figure 2). Initially, PAs bind to PAI-1 through several exosite interactions, defined as secondary interactions between regions outside of the PA active site and the PAI-1 P1-P1′ reactive center (47, 53). The nature of this Michaelis complex is now well-understood from the X-ray structure determination of PAI-1 in complex with active-site mutants of tPA (tPA-S478A) and uPA (uPA-S195A) (47, 53). Through flexible loops on their surface, PAs contact several exosites adjacent to the RCL to facilitate the initial docking step and, in addition to P1 and nearby residues in the RCL, confer proteinase specificity. By forming tight interactions, exosites stabilize the Michaelis complex and lock the PA into a particular orientation to warrant optimal positioning of the P1 residue in the active site of the PA. Furthermore, these additional interactions slow down the dissociation of the PA from its initial docking site, allowing the PA active site serine to attack the P1-P1′ bond to form a tetrahedral intermediate with PAI-1 (54). Successful cleavage of this bond yields the acyl-enzyme intermediate (E~I) in which the PA is covalently linked to the main chain carbonyl of the P1 residue in PAI-1. Following a branched pathway mechanism, the PAI-1/PA reaction is directed either into the inhibitory or into the substrate pathway.

In the *inhibitory pathway* (Figure 2), the formation of the acyl-enzyme intermediate is coupled to a rapid and full insertion of the N-terminal part of the RCL (P16-P1) as strand 4 into the central β-sheet A (s4A) (54). This major conformational change coincides with a 70 Å translocation of the bound PA to the opposite side of the PAI-1 molecule. There, a large part of the PA, including the active site, is deformed by compression against the body of PAI-1. As a result, hydrolysis of the acyl-enzyme intermediate is prevented and the PA remains trapped as a stable PAI-1/PA inhibitory complex (E-I) (49, 55). This mechanism of inhibition was demonstrated by the crystallographic structure of the α_1_-antitrypsin/trypsin complex (49), which is in line with the results from studies that investigated serpin exosite distortion by using nuclear magnetic resonance (56, 57) and circular dichroism (58) studies. In this serpin-protease complex (49), trypsin shows a high degree of conformational disorder as compared to its native form, i.e., a loss of structure for ~37% of the protease. Furthermore, the active site of trypsin is disrupted as Ser195 of the catalytic triad is moved away from its catalytic partners. Several regions in PAI-1 are crucial for the orchestration of loop insertion and are furthermore involved in the energetical coupling of this favorable conformational change to the energy-demanding process of PA distortion (Figure 1) (43). Upon cleavage of the P1-P1′ bond, the PA dissociates from its initial docking site on PAI-1 while releasing the distal P′ side of the cleaved RCL from the PA active site cleft. Simultaneously, the breach region at the top and the shutter region near the center of β-sheet A open up to accommodate the RCL as s4A. The hinge region that contains a conserved series of small hydrophobic residues (P15-P9) initially inserts into the breach region and is a prerequisite for rapid loop insertion. Whereas, the surface-exposed RCL only makes a few contacts with the serpin body, it now becomes an integral part of the central β-sheet A. Further insertion of the RCL, however, is obstructed by a steric clash with hF that is located across β-sheet A. Experimental data favor the hypothesis that hF plays an essential role in PA inhibition by (I) being actively displaced until the loop is fully inserted and the PA has passed to the very end of β-sheet A and (II) by temporarily storing the energy derived from loop insertion in order to distort the PA upon return of hF to its original position, ultimately leading to the formation of the irreversible inhibitory complex (59). Through basic residues in hD and hE in the flexible joint region of PAI-1, these PAI-1/PA complexes bind certain receptors of the low-density lipoprotein receptor (LDLR) family, including low-density lipoprotein receptor-related protein-1 (LRP1), leading to endocytosis and degradation of the complex (60, 61).

In the *substrate pathway* (Figure 2), the acyl-enzyme intermediate is hydrolyzed prior to PA distortion, resulting in the release of regenerated PA (E) from cleaved RCL-inserted PAI-1 (I^*^) (48, 62). This substrate behavior has been associated with the pre-existence of a conformational distinct substrate-like subset of PAI-1 (63, 64), or results from a change in the kinetic parameters that define the partitioning between both branches of the PAI-1/PA reaction (65, 66).

### Factors Influencing the PAI-1/PA Reaction

Several factors have been established that determine target proteinase specificity or influence the partitioning between the inhibitory and substrate branch of the PAI-1/PA reaction pathway. The first region to determine target specificity of PAI-1, and serpins in general, consists of residue at the P1 position and the immediately adjacent residues in the RCL. Indeed, by replacing residues P3-P3′ of the PAI-1 RCL with the corresponding residues of another serpin antithrombin III, this PAI-1 mutant acquired thrombin inhibitory properties (67). Interestingly, vitronectin was shown to alter PAI-1 specificity by also enhancing PAI-1 reactivity toward thrombin in a dose-dependent manner (66, 67). Importantly, studies using PAI-1/serpin chimeras, in which the RCL was replaced with that of other serpins, showed that all chimeras were still effective inhibitors of both PAs, and thus strongly suggested a major contribution of regions outside of the RCL to differences in specificity (68). Based on the crystal structure of the PAI-1/PA Michaelis complexes, interactions between several complementary electrostatic surfaces on PAI-1 and the PAs, referred to as exosites, have been identified (47, 53). One particular region of PAs has been shown to make strong and extensive interactions with PAI-1. The positively charged 37-loop of PAs contacts (I) a negatively charged patch comprising residues in s1B, s2B, and the s3B-hG loop, and (II) the P4′ (Glu350) and P2′ (Ala348) residues in the RCL. This 37-loop/exosite interaction has been proven necessary to ensure the rapid and high-affinity association between PAI-1 and PAs in studies using 37-loop mutants or antibodies specifically binding to this region on the PA molecule (69–71). Furthermore, the residues in the 37-loop of tPA that are responsible for the direct interaction with PAI-1 are less charged in the 37-loop of uPA, resulting in a stronger exosite interaction of PAI-1 to tPA (72). These stronger exosite interactions result in a twice as large contact area of tPA with PAI-1 when compared to uPA, and provide a rationale for the difference in second-order inhibitory rate constants between the two PAs (47, 53).

As mentioned, the importance of several regions within the PAI-1 molecule for RCL insertion as well as for its interaction with the target PAs has been extensively studied. Ample evidence suggested that the nature of the amino acids and the flexibility of these segments are crucial for PAI-1 functionality and that changes made within these regions result in an altered reaction mechanism. Mutations within the hinge region of the RCL (residues P14-P8) altered the specificity toward tPA or uPA and moreover caused PAI-1 to behave predominantly as a substrate toward PAs (65, 73, 74). Stable substrate behavior without any detectable inhibitory activity was also conferred upon a PAI-1 deletion mutant lacking hF and the hF-s3A loop (75). Since both regions are involved in RCL insertion, i.e., the hinge region is initially inserted and hF coordinates the final insertion step, mutations in these regions may change the initial conformation of PAI-1 or impair the kinetics of RCL insertion. Therefore, these changes may ultimately result in the hydrolysis of the acyl-enzyme intermediate (76). Apart from these PAI-1 mutants, the behavior of PAI-1 as an inhibitor or as a substrate has also been reported to be influenced by external conditions, such as low temperature and non-ionic detergents, and ligands (77–79).

### Functional Stability of PAI-1

#### PAI-1 Spontaneously Converts Into an Unreactive Latent Form

Unlike other serpins, PAI-1 has the unique ability to spontaneously convert into a thermodynamically stable latent form with a half-life of ~2 h at 37 °C *in vitro*. This active-to-latent transition occurs by slowly self-inserting the N-terminal part of the RCL into the core of the protein, thereby making the P1-P1′ bond inaccessible for PAs (80). Spontaneous latency transition is rather exceptional and is reported in only a few other serpins (81–83). Several lines of evidence indicate that latency transition in PAI-1 is evolutionarily conserved (84, 85). Therefore, it suggests an important role in auto-regulation of PAI-1 activity to reduce the risk of thrombosis due to the otherwise prolonged antifibrinolytic action of PAI-1. Based on the structures of active and latent PAI-1, the dynamical mechanisms involved in the active-to-latent transition were simulated using a computational approach, and could later be supported by experimental evidence (72, 86, 87). In a concerted manner, strand 1 (s1C) is peeled away from β-sheet C and allows the RCL to move around the gate region while it partially inserts up to P11 (Ser336) in the central β-sheet A that opened up at the breach and shutter region to form s4A. To reach this prelatent state, for which experimental evidence indicates that it co-exists with active PAI-1 in solution (88–91), a change in the bend in hA is required. After being held for an extended period in the prelatent form, full insertion of the final P6-P4 residues of the RCL is blocked by steric clashes between the RCL and the hF-s3A loop that overlies β-sheet A, posing a high-energy barrier. Similar to the conformational changes required for PA translocation, the favorable energy that is released upon partial insertion of the RCL is temporarily stored by an outward movement or unfolding of hF to enable full RCL insertion. Finally, hF returns to its native position across β-sheet A and irreversibly locks PAI-1 in its unreactive latent state. Whereas, during the inhibitory reaction with PAs energy can be recovered to distort the active site of the PA, latency transition is an energetically silent process. Therefore, it has been hypothesized that the energy gain from the favorable insertion of the RCL is used to extract s1C from β-sheet C and to position it alongside the PAI-1 molecule (92). Even though this transition is generally considered to be irreversible, limited reactivation by an unknown mechanism may occur *in vivo* (93). *In vitro*, the inhibitory properties can be restored by treating latent PAI-1 with denaturants followed by refolding (94).

#### *In vivo* Stabilization

*In vivo*, the active form of PAI-1 is stabilized at least 2-fold by the high-affinity association (K_D_ ~ 0.1–1 nM) with the glycoprotein vitronectin that is abundant in plasma and the extracellular matrix (Figure 2) (95–99). The interaction between PAI-1 and vitronectin has been extensively characterized by mutagenesis and competition experiments using monoclonal antibodies, PAI-1/PAI-2 chimeras, and (synthetic) peptides (100–108). Based on these results, the N-terminal somatomedin B (SMB) domain within vitronectin and the flexible joint region, defined by hE, hF, and s1A, within PAI-1 were identified as the primary regions to engage in the interaction. Later, the crystal structure of PAI-1 in complex with the SMB domain of vitronectin (PDB ID 1OC0) provided additional details on the interaction interface, restricting their respective binding sites to the central region of the SMB domain (residues 10–30) and residues in the hE-s2A loop (Arg101), in hE (Pro111 and Phe114), hF (Asp138, Ile135, and Trp139) and s1A (Thr120, Lys122, Gln123, Val124, and Asp125) of PAI-1 (51). Through allosteric modulation of several regions remote from the SMB binding site, vitronectin causes a strong and widespread stabilization of the lower half of the PAI-1 molecule, including hB, hC, hD, hI, and the hI-s5A loop, and induces conformational changes in the RCL without compromising the ability of PAI-1 to associate with PAs (99, 109, 110). By reducing the structural flexibility, binding of vitronectin interferes with the sliding movement that is required to open up the shutter region, and consequently decreases the rate of RCL insertion that ultimately slows down the transition to latent PAI-1. Alternatively, expansion of β-sheet A due to loop insertion during latency transition or during the interaction with PAs results in the dissociation of vitronectin from inactive PAI-1 (95, 111, 112). Apart from the primary high-affinity PAI-1 binding site in the SMB domain, there is experimental evidence for additional PAI-1 binding sites in vitronectin. These sites, comprising a cluster of basic amino acids (residues 348–370 of vitronectin) in the C-terminal region of vitronectin (111–115) as well as the region connecting the SMB domain to the remainder of the vitronectin molecule (residues 111–121 of vitronectin) (116), have been shown to bind PAI-1 with a lower affinity and promote the assembly of higher-order PAI-1/vitronectin complexes (114, 115, 117).

One of the major acute-phase proteins, α_1_-acid glycoprotein, has also been shown to bind and stabilize the active form of PAI-1. Extensive binding studies allowed to identify a binding region that is distinct from that of vitronectin. This α_1_-acid glycoprotein binding region resides in the hI-s5A loop, comprising residues Arg300–Asp305 located at the bottom of PAI-1 β-sheet A (118). Even though this interaction occurs at a slower rate and is less stable as compared to the interaction with vitronectin, it might contribute to the PAI-1-mediated effects during inflammation or acute phase reactions (119).

#### PAI-1 Mutants With Increased Stability

Since its discovery, a vast amount of PAI-1 variants has been generated by both site-directed and random mutagenesis (120). These mutants have been employed in order to gain insights into the structure/function relationship in PAI-1, to identify regions that are important for its biological interactions, and to investigate its pleiotropic functions in various pathological processes. Due to its conformational flexibility, structural studies have benefited in particular from the generation of PAI-1 variants of which the functionally active form is stabilized. While single substitutions cause only a moderate stabilization of PAI-1, the combination of multiple mutations often results in a markedly enhanced stability with half-lives up to 450 h (85). Alternatively, the active conformation can also be maintained by introducing Cys-residues to crosslink flexible regions in PAI-1 that are crucially involved in latency transition (121, 122). This way, PAI-1 variants have been generated in which hD of the flexible joint region is connected to the N-terminal part of hA (engineered disulfide bridge between Val8Cys and Ala74Cys), in which s3A and s5A in the shutter region are covalently linked (Gln169Cys–Gly324Cys), in which the N-terminal part of the RCL is connected to the top of s3A in the breach region (Gln174Cys–Gly332Cys), or in which a combination is used. However, the introduction of only one single disulfide bond at the breach region is sufficient to most effectively preclude latency transition (very long half-life PAI-1, t_1/2_ > 700 h) without affecting its structure (122).

As mentioned, several crystal structures of PAI-1 in its alternative conformations (active, latent, and cleaved PAI-1) or of PAI-1 in complex with biological ligands have been determined by employing these stabilized active mutants (Figure 2 and Table 1). The first stable mutants to be successfully crystallized in the active conformation were the quadruple mutant PAI-1-N150H-K154T-Q319L-M354I, commonly referred to as PAI-1 14-1B (t_1/2_ ~ 145 h) (123, 133) and a variant harboring a fifth mutation, PAI-1-N150H-K154T-Q301P-Q319L-M354I, referred to as PAI-1-stab (46, 134). Later, the structure of active PAI-1-W175F (t_1/2_ ~ 7 h) was resolved as well (124). Apart from its prolonged half-life, PAI-1-W175F behaves similarly to wild-type PAI-1 and is therefore a more valid representative of wild-type PAI-1. Comparison of the available PAI-1 14-1B and PAI-1-W175F structures revealed numerous structural differences, with the most prominent one located in the region containing hF and the hF-s3A loop. Three of the mutations in PAI-1 14-1B and PAI-1-stab are clustered in and below the hF-s3A loop (Asn150His, Lys154Thr, and Gln319Leu) and induce a 3_10_-like helix covering residues 151–157 that connects hF to the underlying β-sheet A through a hydrogen-bonding network. As a consequence, the energy barrier for hF displacement during the final step in RCL insertion is raised, explaining both the stabilization of the active conformation as well as the increased substrate behavior upon interaction with PAs that is observed for PAI-1 14-1B and PAI-1-stab. In contrast, the stabilization caused by the single amino acid substitution of the conserved tryptophan in PAI-1-W175F appears to be the result of local effects in the breach region that restrict initial loop insertion (124, 135).

**Table 1:** List of X-ray crystallographic structures containing human PAI-1 available in the Protein Data Bank (PDB).

**Form**	**PDB ID**	**PAI-1 variant**	**Ligand**	**Resolution (Å)**	**References**
Active	1B3K	PAI-1 14-1B^a^	NA	2.99	(123)
	1DB2	PAI-1-stab^b^	NA	2.70	(46)
	1DVM	PAI-1 14-1B	NA	2.40	(45)
	3Q02	PAI-1-W175F	NA	2.30	(124)
	3R4L	VLHL-PAI-1^c^	NA	2.70	(122)
Latent	1C5G	PAI-1-wt^d^	NA	2.60	(125)
	1DVN	PAI-1 14-1B	NA	2.10	(45)
	1LJ5	PAI-1-wt	NA	1.80	–
	3Q03	PAI-1-W175F	NA	2.64	(124)
Cleaved	9PAI	PAI-1-A335P	NA	2.70	(62)
	3CVM	PAI-1 14-1B	NA	2.03	(126)
	3EOX	PAI-1-stab	NA	2.60	(48)
+ Ligand	1OC0	PAI-1 14-1B	SMB domain of vitronectin	2.28	(51)
	3PB1	PAI-1 14-1B	Catalytic site mutant of uPA, uPA-S195A	2.30	(53)
	5BRR	PAI-1 14-1B	Catalytic site mutant of tPA, tPA-S195A	3.16	(47)
+ Inhibitor	1A7C	PAI-1-A335E	RCL-derived inhibitory peptide P14-P10	1.95	(127)
	4AQH	Latent PAI-1 14-1B	AZ3976	2.40	(128)
	3UT3	PAI-1 14-1B	Embelin	2.42	(129)
	4IC0	PAI-1 14-1B	Gallate	2.32	(130)
	4G8O	PAI-1 14-1B	CDE-096	2.71	(131)
	4G8R	PAI-1 14-1B	CDE-096	2.19	(131)
	6I8S	PAI-1 14-1B	Fab^e^ fragment of MEDI-579	2.90	(132)
	5ZLZ	PAI-1 14-1B	PAItrap 2	3.58	–
	6GWN	PAI-1-W175F	Nanobody Nb42 and Nb64	2.03	(52)
	6GWP	PAI-1-stab	Nanobody Nb42 and Nb64	2.28	(52)
	6GWQ	PAI-1-stab	Nanobody Nb42	2.32	(52)
	6ZRV	PAI-1-W175F	Nanobody Nb93	1.88	(41)

a *PAI-1-N150H-K154T-Q319L-M354I*.

b *PAI-1-N150H-K154T-Q301P-Q319L-M354I*.

c *Very long half-life PAI-1, PAI-1-Q174C-G332C*.

d *Wild-type PAI-1*.

e *Antigen-binding fragment*.

*NA, not applicable*.

#### External Conditions Affecting PAI-1 Stability

Apart from being stabilized through interactions with its physiological ligands, several external conditions have been shown to affect the rate of latency transition in PAI-1 *in vitro*. During the search for the optimal purification conditions of recombinant PAI-1, a low temperature (4°C), a low pH (~5.5), and a high salt concentration (1 M NaCl) contributed to increased PAI-1 stability (98). Since a decrease in pH causes protonation of imidazole groups, it was suggested that one or more histidine residues might be directly responsible for the pH-dependent stability of PAI-1. It was first speculated that His^143^, localized at the top of hF, might be responsible for this effect (136). However, site-directed mutagenesis studies could only demonstrate a direct role for His^364^, situated on the C-terminal end of s4B in close vicinity to hD in the flexible joint region and to the W86-loop (137). The salt stabilization was further investigated based on the observation of an anion-binding site in a crystal structure of PAI-1 14-1B in the active conformation (45). It was suggested that by forming close interactions with partially positive nitrogen residues on each side of the anion-binding site, i.e., with Lys323 and Lys325 in β-sheet A and Ser149 and His150 in the hF-s3A loop, chloride binding increases the energy barrier of the final stage in latency transition. Also, a more pronounced stabilization was correlated with an increased electronegativity of the anion (F^−^ ≥ Cl^−^ > Br^−^ > I^−^), resulting in tighter interactions. Notably, the proposed anion-binding site is located in the hF-s3A loop that is structurally different in PAI-1 14-1B as compared to “wild-type” PAI-1-W175F. Indeed, anion-binding could not be observed within this region in the more recent structure of active PAI-1-W175F (124) and is thus likely to be an artifact resulting from the induced conformational changes in the hF-s3A loop region in PAI-1 14-1B. However, a previously unknown chloride-binding site centered in the gate region could be unambiguously identified. This led to a revised hypothesis of the salt stabilizing effect on PAI-1 inhibitory activity, i.e., delaying latency transition by blocking the gate through bridging of several structural elements located between the s3C/s4C loop and the hG-s3B loop. Also, the preferential stabilization of other halide salts could not be extended to PAI-1-W175F or wild-type PAI-1, which were most dramatically stabilized by sodium chloride yielding half-lives well above 30 h at a 1 M salt concentration (124). Interestingly, two zinc-binding sites could clearly be identified within the same crystal structure of PAI-1-W175F. Since the metals appeared at the interface between two PAI-1 molecules inside the crystal, it remained debatable whether one or both binding sites are physiologically relevant. However, one zinc ion was strongly coordinated by N-terminal His2 and His3 (124). Almost simultaneously it was shown that type I metal ions (calcium, magnesium, and manganese) have modest stabilizing effects on PAI-1 activity, whereas type II metals (cobalt, copper, and nickel) had a more pronounced effect, either destabilizing PAI-1 in the absence of vitronectin or adding up onto the stabilization caused by simultaneous binding of vitronectin (138). Even though competitive binding experiments suggested that these effects were mediated through a single metal-binding site (139), a copper-binding site involving N-terminal His2 and His3 was identified that only accounted for the stabilizing, and not the destabilizing, effect of copper (140). The existence of a second copper-binding site has been further confirmed by the observation that copper facilitates an early step in PAI-1 latency transition by increasing protein dynamics in the flexible joint region and the helices underlying the shutter region, which could also be observed when copper bound to a mutant lacking His2 and His3 (141). Apart from salts and metals, high concentrations of arginine have been shown to elute subendothelial matrix-bound PAI-1 and to specifically stabilize the PAI-1 active conformation (142, 143). Since arginine residues are clustered in the stretch of basic residues in the C-terminal region of vitronectin, free arginine might contribute to an enhanced PAI-1 stability in a similar way as the arginine-rich C-terminal region of vitronectin.

## (PATHO)Physiological Roles of PAI-1

As the major physiological inhibitor of plasminogen activators tPA and uPA, PAI-1 plays a regulatory role in the fibrinolytic system by controlling plasmin formation. Not only is the plasminogen activator/plasmin system involved in fibrinolysis, it has also a profound role in multiple physiological processes, including the degradation of extracellular matrix (ECM), tissue remodeling, wound healing, angiogenesis, cell migration, and inflammation (144). Upon uPA-mediated activation of plasminogen, either by two-chain uPA or single-chain uPA bound to the uPA receptor (uPAR), plasmin can degrade several ECM components either directly or indirectly through the activation of matrix metalloproteases (MMPs). Degradation of the ECM may then facilitate cell invasion into the surrounding tissue. Furthermore, by increasing the availability of growth factors, such as vascular endothelial growth factor, fibroblast growth factor, and transforming growth factor-β, the role of plasmin further extends to the control of angiogenesis, cell growth, and cell differentiation. Importantly, independent of its effect on plasmin formation, PAI-1 directly interacts with specific matrix components, including vitronectin, LRP1, and the uPA/uPAR complex to affect cell migration and intracellular signaling.

The (patho)physiological role of PAI-1 has been extensively studied by comparing the phenotype observed in human PAI-1 deficiency with that of mice engineered to be completely PAI-1 deficient by gene targeting (PAI-1^−/−^ mice). In humans, PAI-1 deficiency is an uncommon disorder that can be caused by mutations in the *SERPINE1* gene leading to the production of non-functional PAI-1 protein (145) or by a complete absence of PAI-1 plasma antigen (146–148). Typically, this disorder is characterized by mild to moderate bleeding in response to injury, trauma, or surgery. In women, PAI-1 deficiency may cause severe blood loss during menstruation and pregnancy-related complications, such as prepartum bleeding, preterm labor, or miscarriage (149–151). PAI-1^−/−^ mice were shown to be viable, fertile, and developed normally (152). Furthermore, disruption of the PAI-1 gene did not appear to impair hemostasis, but was associated with increased resistance to thrombosis and with a milder hyperfibrinolytic state as compared to humans (153). In contrast to PAI-1 deficient mice, several lines of transgenic mice overexpressing native or stabilized PAI-1 of human and murine origin have been established. These lines have been generated to explore the effects of elevated PAI-1 levels on, e.g., the progress of thrombosis (154, 155), pulmonary fibrosis (156), and obesity (157). Furthermore, these transgenic mice often display hair loss and skin abnormalities. Importantly, transgenic mice expressing a reactive site inactive PAI-1 mutant exhibit complete phenotypic rescue, while transgenic mice expressing PAI-1 with reduced affinity for vitronectin manifest all of the phenotypic abnormalities, underscoring the fact that PAI-1 affects physiological processes by acting through multiple pathways (158). In humans, two variations in the promoter region of the PAI-1 gene occur frequently and have been shown to affect PAI-1 levels (159–161). Firstly, the 4G/5G polymorphism refers to a single guanosine insertion/deletion at position 675 upstream of the transcription site (159). It has been suggested that the 4G allele is associated with higher PAI-1 activity since the 5G allele harbors an additional binding site for a transcriptional repressor. Secondly, the G/A polymorphism is characterized by a single nucleotide substitution of guanine for adenine at position 844 upstream of the transcription site, generating a consensus binding sequence for transcription factor Ets-1 which increases the transcription rate (161). Taken together, the association of these PAI-1 gene polymorphisms and/or elevated PAI-1 levels with several pathologies have been extensively studied in both humans and in PAI-1 deficient or transgenic mice.

### PAI-1 in Cardiovascular Disease

Elevated levels of PAI-1 downregulate tPA and uPA activity and create a prothrombotic or hypofibrinolytic state that was suggested to contribute to the pathogenesis of cardiovascular diseases (CVD) (162). As mentioned, several lines of transgenic mice that overexpress PAI-1 have been developed and support a contribution of elevated PAI-1 levels to thrombosis and CVD. The first line of transgenic PAI-1 mice overexpressed native human PAI-1 and was shown to develop transient venous thrombosis in the tail and hind limbs and subcutaneous hemorrhage (154). Later, human PAI-1-stab mice were generated that displayed age-dependent coronary arterial thrombosis and myocardial infarction (155). In contrast, spontaneous thrombosis could not be observed in transgenic mice that overexpress stabilized murine PAI-1 (163). However, it should be noted that (I) the choice of the promoter that drives PAI-1 expression, (II) the nature of the stable variant, and (III) a cross-species difference in PAI-1 function may have contributed to the observed phenotypic differences. In humans, many studies have suggested that PAI-1 gene polymorphisms, possibly leading to higher PAI-1 levels, are an independent risk factor for major adverse cardiovascular events (MACE) including myocardial infarction (MI) (164–167) and ischemic stroke (168), as well as coronary heart disease (CHD) (169), venous thrombosis (170–172), and atherosclerosis (173). However, despite the observed link in these studies, these findings are in contradiction with other available data (174–178). Similarly, independent of the contribution from PAI-1 gene polymorphisms, ample evidence has been provided of a link between elevated PAI-1 levels and MI or stroke (179–181), CHD (182), venous thrombosis (183), and atherosclerosis (184, 185). In a recent systematic review of all studies published between 1991 and 2016 that examined the association of PAI-1 with MACE (defined as death, MI, and stroke) or restenosis (the recurrence of treated coronary artery stenosis), Jung et al. substantiated a link between elevated plasma PAI-1 antigen levels, but not PAI-1 activity levels, and MACE in both incident and secondary event populations (181). MI is most often a result of CHD and is caused by the disruption of an atherosclerotic plaque, thereby exposing a procoagulatory surface of the coronary vessel that gives rise to occlusive thrombus formation (186). Several studies have reported elevated PAI-1 levels in several cell types associated with atherosclerotic plaques in human coronary arteries, including endothelial cells, vascular smooth muscle cells (SMCs), and macrophages (184, 187, 188). In mice, PAI-1 deficiency has been shown to be protective (189) or promoting (190) in the development of atherosclerosis, however, no effect of PAI-1 on atherosclerosis has been observed as well (191). Indeed, overproduction of PAI-1 in a diseased vessel wall may contribute to the progression of atherosclerosis by reducing local plasmin production which is physiologically required for the removal of fibrin, ECM remodeling, and SMC proliferation. However, when the controlling effect of PAI-1 on plasmin formation is abolished, it may contribute to the atherogenic role of plasmin, as plasmin is also involved in lipoprotein modification, macrophage cholesterol accumulation, inflammation, and foam cell formation (192, 193). Furthermore, PAI-1 has been shown to have an ambiguous role in neointima formation (194–198). In this respect, in the same systematic review by Jung et al., low PAI-1 antigen and activity levels were associated with increased restenosis, highlighting the complex role of PAI-1 in vascular remodeling (181). Despite the links provided between PAI-1 and CVD, certain studies could not confirm these associations or the significance was lost after adjustment for other risk factors (199–201). A positive correlation has been demonstrated for plasma PAI-1 levels and known risk factors for developing CVD, including age, sex, obesity, hyperlipidemia, insulin resistance, and diabetes (162, 181, 202, 203). Age is an important risk factor for most chronic diseases including cardiovascular disease, type 2 diabetes, and metabolic syndrome. Furthermore, PAI-1 levels have been reported to increase with age in various tissues. More recently, PAI-1 has been identified not only as a marker but also as a mediator of cellular senescence associated with aging and aging-related pathologies (204).

## Diverse Approaches To Inhibit PAI-1

As PAI-1 is considered a risk factor in various pathological conditions, many efforts have been devoted to the development of PAI-1 inhibitors, including small molecules, synthetic peptides, RNA aptamers, monoclonal antibodies (mAbs), and antibody derivatives. Whereas, some marketed drugs have been shown to attenuate PAI-1 synthesis or secretion (205), the majority of PAI-1 inhibitors currently in development can influence PAI-1 functionality in at least three possible ways: (I) by directly blocking the initial formation of the Michaelis complex between PAI-1 and PAs, (II) by preventing the formation of the final inhibitory complex, resulting in substrate behavior of PAI-1, or (III) by accelerating the active-to-latent transition of the PAI-1 molecule or an otherwise inert form. Despite extensive *in vitro* and *in vivo* characterization, no PAI-1 inhibitor is currently approved for therapeutic use in humans. This is mainly due to affinity and specificity issues, which are especially observed for small molecules. Furthermore, the structural plasticity of PAI-1 and the possible counteraction of PAI-1 binding partners, such as vitronectin, pose a real challenge to develop PAI-1 inhibitors that retain their capacity to modulate PAI-1 activity *in vivo*. To improve their properties or to guide the rational design of novel PAI-1 inhibitors it is essential to get a deeper understanding of PAI-1 inhibition at the molecular level. In addition to the several crystal structures of PAI-1 in active, latent, or cleaved conformation (45, 48, 124), a handful of structures containing PAI-1 in complex with inhibitory peptides, small molecules, and antibody fragments have been described (Table 1). Furthermore, by using a broad range of biophysical and biochemical methods, including competitive binding experiments, mutagenesis, and computational docking, the presumptive binding regions of the majority of PAI-1 inhibitors have been mapped and can be related to the mechanisms by which they interfere with PAI-1 functionality.

### Synthetic Peptides

#### RCL-Mimicking Peptides

Insertion of the RCL into the central β-sheet A is a crucial step in the inhibitory mechanism of serpins in order to translocate and irreversibly trap the target proteinase. In this regard, synthetic peptides derived from the RCL-sequences of antithrombin III and α_1_-antitrypsin were shown to convert the respective serpins from an inhibitor to a substrate. By binding between s3A and s5A in β-sheet A and thus becoming s4A, these peptides prevent endogenous RCL insertion upon interaction with the target proteinase, resulting in cleavage of the serpin and release of regenerated proteinase (206). Taking a similar approach, several peptides that mimic different fragments of the RCL of PAI-1, such as P14-P1 (207), P14-P10 (127), P14-P9 (208), P14-P7 (136), and P8-P3 (208), were designed and evaluated for their PAI-1 modulating properties. The first peptide, corresponding to residues P14-P1 of the RCL, was shown to rapidly inhibit PAI-1 activity by accelerating the conversion to a non-reactive PAI-1 form and effectively enhanced *in vitro* fibrinolysis in both platelet-poor and platelet-rich clots (207). However, when PAI-1 was bound to its biological cofactor vitronectin, the PAI-1 neutralizing effects of this peptide were considerably reduced. A comparable mechanism was observed for the P8-P3 peptide that mimics the C-terminal part of the RCL that inserts at the bottom of the β-sheet A in latent PAI-1. Unlike peptides P14-P1 and P8-P3, peptides P14-P7, P14-P10, or P14-P9 that correspond to the N-terminal part of the RCL converted PAI-1 from an inhibitor to a substrate for tPA (127, 136, 208). By showing that binding of P14-P7 and the formation of latent PAI-1 are competitive events, the first evidence was provided for a binding site in the central β-sheet A cleft (136). The high-resolution crystal structure of PAI-1 mutant PAI-1-Ala335Glu in complex with two P14-P10 peptides (PDB ID 1A7C) further confirmed this presumption (127). The structure revealed that both peptide molecules bound inside the cleft between s3A and s5A, with the first molecule occupying the same space as RCL residues P14-P10 in latent and cleaved PAI-1, and the second one occupying the same space as residues P6-P2 in cleaved PAI-1. Since the different effects of RCL-derived peptides on the outcome of the PAI-1/PA reaction, i.e., conversion to either inert or substrate PAI-1, did not seem compatible with one common binding position inside the β-sheet A cleft, it was finally suggested that peptides mimicking the C-terminal part of the RCL (P8-P3) act by accelerating the irreversible transition to an inert form of PAI-1, whereas peptides that mimic the N-terminal part of the RCL (P14-P9) primarily induce PAI-1 substrate behavior (208).

#### Other Peptides

A few other peptides have been isolated from a phage-display peptide library including paionin-1 (209), which does not impair PAI-1 activity, and paionin-4 (210), which accelerates the active-to-latent conversion of PAI-1. *In silico* docking of paionin-1 into the crystal structure of PAI-1 suggested a binding site in the flexible joint region that was supported by site-directed mutagenesis and competitive binding of other molecules targeting the same region, such as XR-5118 and bis-ANS. As paionin-1 is able to prevent binding of the PAI-1/uPA complex to LRP1, paionin-1 or other compounds binding in the same region may be of benefit in cases where disruption of the signaling function of uPA/uPAR/LRP1 is desired. Paionin-4 presumably binds PAI-1 at the loop between hD and s2A and is suggested to induce a conformational change that facilitates loop insertion.

### RNA Aptamers

RNA-aptamers are 8–15 kDa single-stranded nucleic acid ligands that tend to bind to highly positive regions on proteins and block protein-protein interactions. In this respect, a few RNA aptamers have been developed in order to interfere with the interactions between PAI-1 and its binding partners. WT-15 and SM-20 are able to disrupt the functional interaction between vitronectin and PAI-1 without compromising the PA-inhibitory function of PAI-1 (211). Expression of these aptamers in human breast cancer cells decreased cell migration and invasion and additionally decreased PAI-1 and uPA levels while increasing the stable PAI-1/uPA complex (212). Other aptamers, R10-4 and R10-2, were able to interfere with the formation of the stable PAI-1/tPA complex but not with the PAI-1/uPA complex, suggesting a binding site not directly involved in PAI-1/PA interactions, without disrupting the PAI-1/vitronectin interaction (213).

### Small Molecules

**Figure 3:**
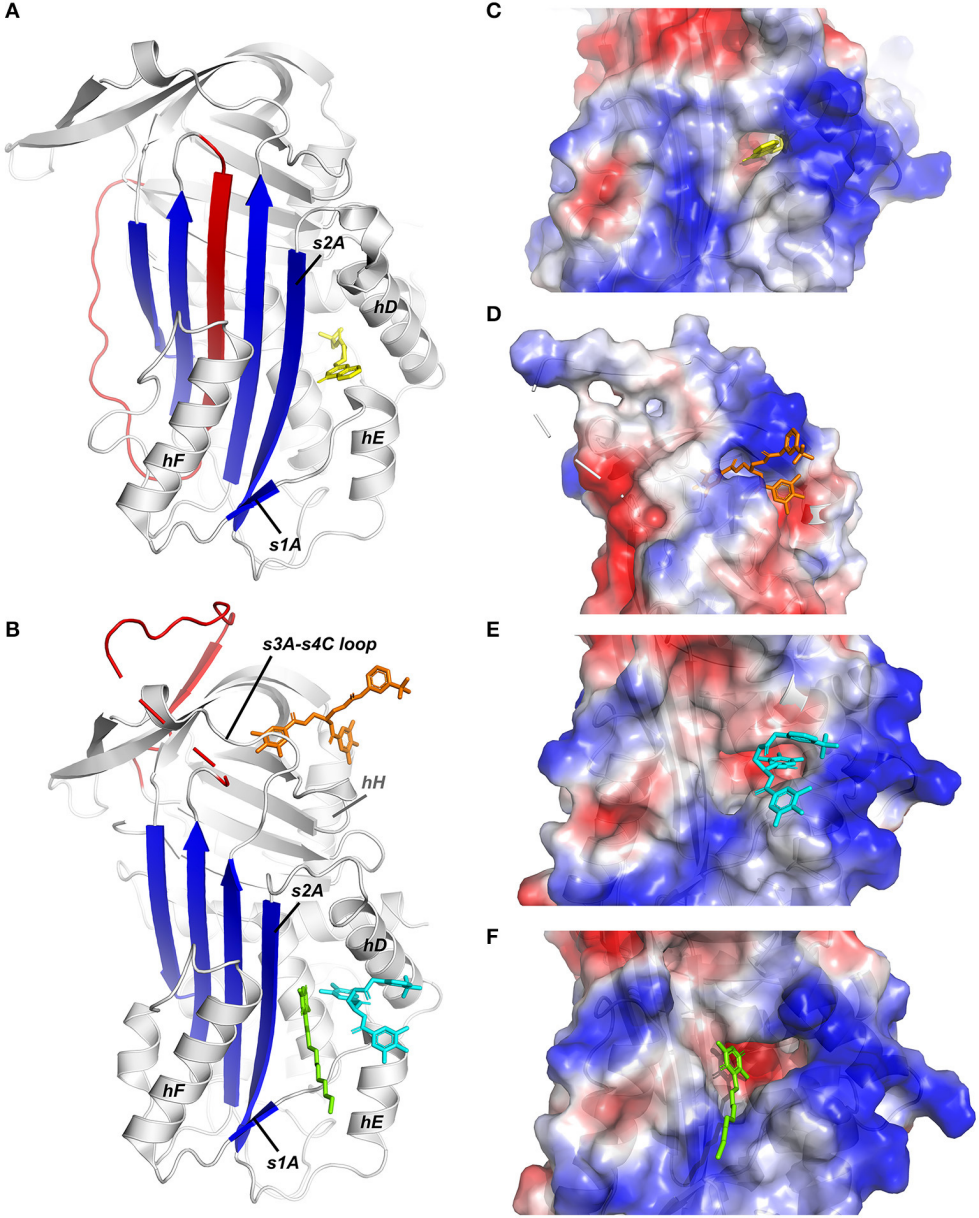
X-ray crystallographic structures of small molecule inhibitors bound to PAI-1. **(A)** Structure of latent PAI-1 in complex with AZ3976 (128). **(B)** Superimposition of the structures of active PAI-1 in complex with embelin (129) and CDE-096 (131). PAI-1 is colored white with the central β-sheet A colored blue and the RCL colored red. Secondary structure elements involved in binding to the compounds are indicated. AZ3976 is colored yellow, and embelin is colored green. CDE-096 in the structures obtained by cocrystallization or crystal soaking is colored orange or cyan, respectively. **(C)** AZ3976 bound to a deep pocket aligned by hD and s2A in latent PAI-1 [PDB ID 4AQH (128)]. **(D)** CDE-096 bound to active PAI-1 obtained by cocrystallization [PDB ID 4G8O (131)]. **(E)** Detail of the structure of CDE-096 bound to active PAI-1 obtained by crystal soaking [PDB ID 4G8R (131)]. **(F)** Detail of the structure of embelin bound a groove aligned by hD, hF, s2A, and the hE-s1A loop in active PAI-1 [PDB ID 3UT3 (129)].

Since the mid-90s, several low molecular weight (LMW) inhibitors possessing a large structural diversity have been described and grouped based on their chemical composition [extensively reviewed by Fortenberry (214) and Rouch et al. (215)]. Lead molecules have been discovered using various approaches, including through isolation from bacterial biomass or a library of natural products, by high-throughput screening (HTS) of synthetic libraries, and by structure-based virtual screening. Subsequently, many of these compounds have been modified based on structure-activity relationship (SAR) studies in order to improve their selectivity and specificity and their inhibitory and physicochemical properties. Even though only a few structures of PAI-1 complexed with small molecules were determined, they provided evidence for a common compound-binding pocket within the flexible joint area of PAI-1 (Figure 3).

The first published crystal structure of a PAI-1/inhibitor complex involved compound AZ3976 (PDB ID 4AQH) (Figure 3C) (128). AZ3976 was identified by HTS of the AstraZeneca compound collection and shown to inhibit PAI-1 activity in *in vitro* chromogenic and clot lysis assays. Titration of PAI-1 with AZ3976 revealed that the compound was only bound to 30% of the total PAI-1 present, corresponding to the non-reactive subpopulation (latent or cleaved) in the preparation of active PAI-1. This was confirmed by affinity data, showing a high binding affinity toward latent PAI-1 whereas no binding was observed toward the active form. Based on the structure of latent PAI-1 complexed with AZ3976, a deep ligand-binding pocket within the flexible joint region was identified with the entry located between hD and s2A. Importantly, this binding site appeared to be more accessible in latent PAI-1, however, tight binding of AZ3976 requires small conformational changes. Since AZ3976 has been shown to accelerate the active to latent transition of PAI-1, it was therefore suggested to bind to a latent-like prelatent form from which latent PAI-1 is then generated more rapidly.

Not much later, a second crystal structure of PAI-1 containing a small molecule inhibitor, embelin, was published (PDB ID 3UT3) (Figure 3F) (129). Embelin was identified as a PAI-1 antagonist by screening a library of natural products. Structural and site-directed mutagenesis results have shown that embelin binds to a small and charged groove aligned by hD, hF, s2A, and the hE-s1A loop in active PAI-1 (129), located adjacent to the larger and deeper pocket in (pre)latent PAI-1 that can be occupied by AZ3976 (128). It was proposed that embelin fixes s2A to the neighboring hD and hE, and thereby interferes with the sliding movement of s2A and s3A into the flexible joint region in order to accommodate RCL insertion. Indeed, consistent with this theory, embelin has been shown to act by a two-step mechanism including a fast reversible step of inducing PAI-1 substrate behavior, followed by a slow irreversible induction of an inactive form. Despite the ability of AZ3976 and embelin to inhibit PAI-1 activity *in vitro*, the presence of vitronectin abolished the capacity of both compounds to bind and modulate PAI-1 activity. Since the binding sites for AZ9376 and embelin partially overlap with the binding site for vitronectin in active PAI-1, the protective effects of vitronectin might be caused by sterically blocking their binding sites or, in the case of AZ3976, by preventing the formation of the prelatent structural state to which AZ3976 preferably binds. Importantly, the binding pocket within the flexible joint region, which has been observed in the crystal structures of PAI-1 complexed with AZ3976 and embelin, is consistent with the binding sites for other small molecules that were determined mainly through competitive binding studies, mutagenesis studies, and molecular modeling. Several negatively [AR-H029953XX (216), ANS, Bis-ANS] and positively [XR5118 (217, 218)] charged amphipathic inhibitors have been shown to bind overlapping but non-identical binding sites within this hydrophobic pocket, resulting in variable induced molecular changes in PAI-1 and in a differential susceptibility to vitronectin-bound PAI-1 (219). First, it was demonstrated that both groups inhibit PAI-1 via a two-step mechanism, involving a rapid reversible conversion into a PAI-1 form exhibiting substrate behavior, followed by a slower irreversible conversion into a non-reactive form. However, a different study showed that both AR-H029953XX and XR5118 induce a direct conversion of PAI-1 into a non-reactive form, possibly due to the differences in compound concentrations that were used to conduct experiments. A concentration-dependent effect could also be observed for tiplaxtinin (PAI-039), which induces PAI-1 substrate behavior at lower concentrations and converts PAI-1 to a non-reactive form at high concentrations (88). Interestingly, several studies reported that PAI-1 polymerization could be induced by negatively charged organochemical inhibitors following conversion to a non-reactive form (219–221).

Apart from this common compound-binding pocket, a third crystal structure of the cocrystallized PAI-1/CDE-096 complex (PDB ID 4G8O) elucidated the binding mode of CDE-096 which is, in contrast to the aforementioned compounds, active against both free and vitronectin-bound PAI-1 (Figure 3D) (131). CDE-096 was synthesized based on a SAR study of a high-affinity polyphenolic PAI-1 inhibitor (222). Structural studies, substantiated with site-directed mutagenesis results, revealed a binding site at the interface composed of residues from the s3A/s4C loop, β-sheets B and C, and hH, referred to as the sB/sC pocket. Using a combination of biochemical experiments, a mechanism of action was proposed that involves reversible allosteric modulation of RCL conformation to block initial PAI-1/PA Michaelis complex formation. Furthermore, although CDE-096 and vitronectin reduce PAI-1's affinity for one another, their binding is not strictly mutually exclusive, suggesting allosteric modulation through reciprocal communication between the high-affinity compound- and vitronectin-binding sites. In crystal soaking studies that require high concentrations of CDE-096 to be incubated with preformed PAI-1 14-1B crystals, a second possible binding site was observed and shows overlap with the binding site for AZ3976 in latent PAI-1 (PDB ID 4G8R) (Figure 3E). However, based on the mutagenesis results and the capacity of CDE-096 to bind both active and latent PAI-1 with similar affinity, it was argued to be an artifact induced by the high concentrations used for crystal soaking.

Another class of small molecules was discovered by virtual screening of a library of commercially available chemicals. To address the lack of efficacy when translated into *in vivo* settings often encountered in high-throughput screening of large compound libraries, two filters were applied representing (I) the general drug-likeliness based on clinically used drug molecules, and (II) the specific lead-likeliness based on the RCL peptide P14-P10 as well as reference inhibitors that bind in the region of the vitronectin binding site in PAI-1 (219, 223). Next, docking simulations for the remaining compounds focused on the cleft in β-sheet A that is occupied by the RCL following loop insertion. TM5007, the most effective compound, exhibited high specificity for the PAI-1/PA system and was furthermore effective in *in vivo* models of thrombosis and fibrosis (223). SAR studies on TM5007 resulted in the selection of TM5275 with an improved inhibitory profile and better oral bioavailability (224). A similar docking simulation suggested that TM5275 binds within the cleft between the strands of β-sheet A, albeit at a different position compared to its precursor TM5007. Whereas, TM5007 docked into the space occupied by P8-P3 in the latent form, TM5275 docked at the P14-P9 position. The differences in their presumed binding sites within the cleft also seemed to correlate with their mechanisms of action, i.e., by either preventing PAI-1/PA complex formation (TM5007) or inducing substrate behavior of PAI-1 (TM5275) (223, 224). Further structure-optimization by substituting the lipophilic moiety and varying the acyl-type linker length led to the discovery of smaller derivatives, including TM5441 and TM5484 (225). Although these compounds have originally been designed to bind the central β-sheet A cleft, there is no experimental evidence that confirms their binding site or their mechanism of action.

Even though many small molecules have been shown to be potent PAI-1 inhibitors *in vitro* or *in vivo*, several other factors, such as the lack of information regarding their exact inhibitory mechanism and/or binding area in PAI-1, the inability to modulate the activity of vitronectin-bound PAI-1, and the possibility to induce PAI-1 polymerization may partially hamper the future rational design of novel effective small molecules.

### Antibodies and Antibody Derivatives

**Figure 4:**
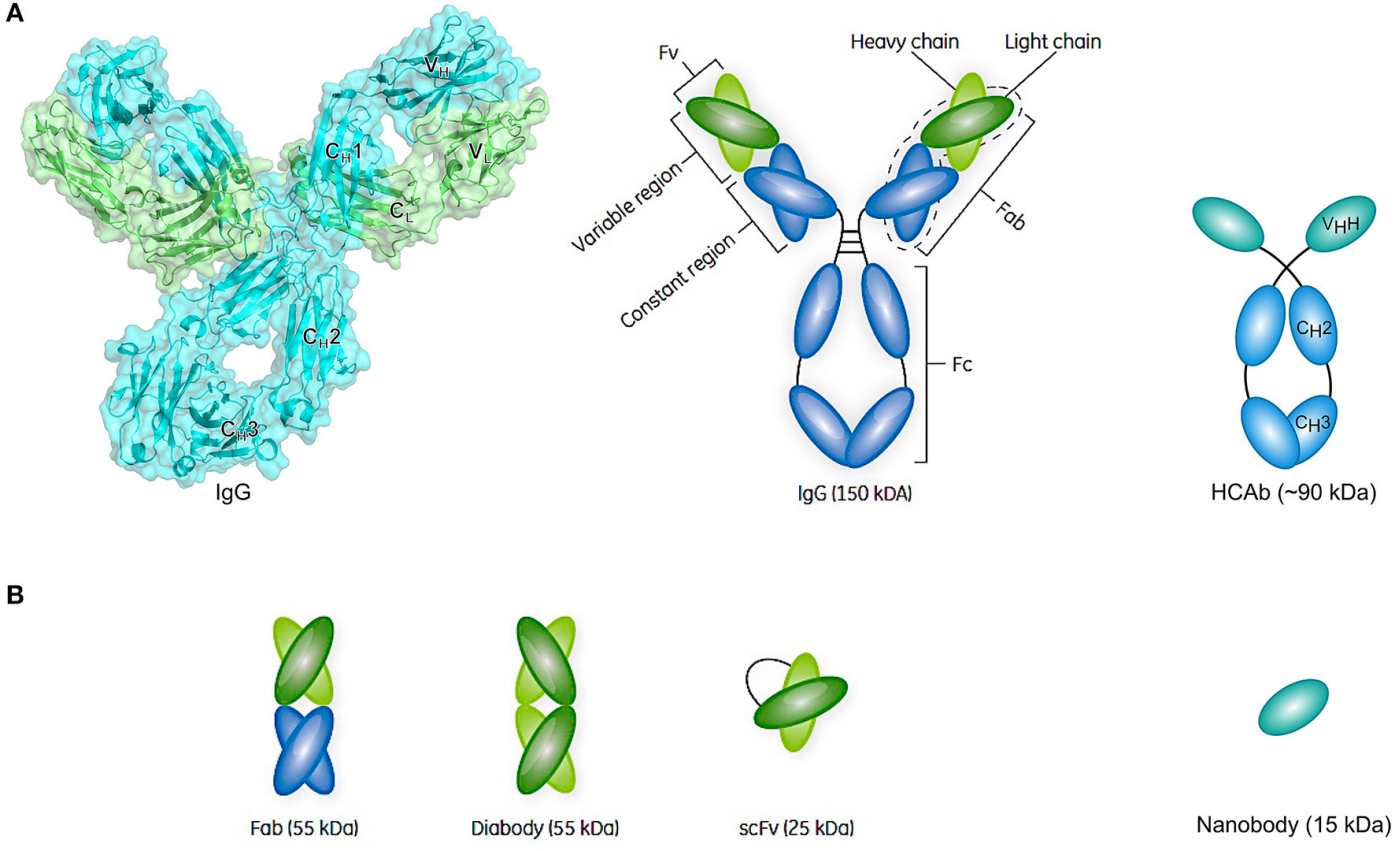
Schematic representation of an antibody structure and different antibody fragment formats. **(A)** The left panel shows the cartoon and biological surface representation of the full-size crystal structure of Pembrolizumab, a human anti-PD1 immunoglobulin G4 (IgG4) antibody [PDB ID 5DK3 (226)]. A conventional antibody is a Y-shaped heterotetrameric glycoprotein consisting of two identical heavy (cyan) and two identical light chains (green). The heavy chain comprises one variable domain (V_H_) and three constant domains (C_H_1, C_H_2, and C_H_3), whereas the light chain comprises one variable (V_L_) and one constant (C_L_) domain. The panel on the right shows a simplified schematic representation of a conventional antibody and a heavy-chain-only antibody (HCAb). Each arm of the conventional antibody represents the antigen-binding fragment (Fab) that comprises the constant region (C_H_1 and C_L_ domains) and the variable fragment (Fv) containing V_L_ and V_H_. The stem of the antibody comprises two copies of the C_H_2-C_H_3 domains that form the crystallizable fragment (Fc). The HCAb comprises two heavy chains, each combining one variable V_H_H domain, referred to as nanobody, and two constant domains (CH2 and CH3). **(B)** Schematic representation of a selection of antibody fragments, including Fab, diabody, single-chain variable fragment (scFv), and nanobody. Figure adapted from Rodrigo et al. (227).

Conventional antibodies are Y-shaped heterotetrameric glycoproteins (150 kDa) composed of two light and two heavy chains that are linked together by multiple disulfide bonds (Figure 4A) (228). Each light chain comprises one variable (V_L_) and one constant domain (C_L_), whereas each heavy chain comprises one variable domain (V_H_) and three constant domains (C_H_1, C_H_2, and C_H_3). The Ig unit can be divided into three functional components, namely two identical antigen-binding fragments (Fabs) and one crystallizable fragment (Fc). In each Fab fragment, the variable fragment (Fv) is responsible for the recognition of and high-affinity binding to a specific antigen and is composed of the variable domains of both chains (V_L_ and V_H_). The amino acid residues of the V-regions that are in direct contact with the antigen are referred to as the paratope, whereas the binding site for the antibody on the surface of the antigen is referred to as the epitope. By connecting the V_H_ and V_L_ domain of a conventional antibody through a flexible polypeptide linker consisting of serines and glycines, a ~25 kDa single-chain variable fragment (scFv) can be created that usually retains the antigen-binding capacity of the parental mAb (Figure 4B). Subsequently, two scFvs can be combined in order to generate a ~55 kDa diabody that can either target the same epitope on another molecule of the same antigen (bivalent), another epitope on the same antigen (biparatopic), or another antigen (bispecific) (Figure 4B).

Later it was discovered that, besides conventional antibodies, the sera of camelids (such as camels and llamas) (229) as well as cartilaginous fish (such as nurse sharks) (230) naturally contain functional heavy-chain-only antibodies (HCAbs) (231). In contrast to conventional antibodies, camel HCAbs (~90 kDa) are devoid of the light chains and the heavy chain C_H_1 domains that normally serve to anchor the light chains (Figure 4A). The V_H_H domain can be isolated from the HCAb and be produced as such at a large scale in bacterial expression systems, ultimately yielding a recombinant single-domain antibody referred to as nanobody (Nb) (Figure 4B). In addition to having high binding affinities toward antigens in the nano- or even picomolar range, Nbs express a favorable stable behavior in harsh conditions, including high temperatures, high protein concentrations, high pressure, and the presence of detergents or denaturants (232, 233). Furthermore, resistance to pepsin or chymotrypsin can be conferred upon nanobodies by introducing an additional disulfide bond, suggesting the possibility of oral administration (234). Furthermore, their small size makes them good candidates when it is required to penetrate dense tissues in order to bind hard to reach targets or to deliver functional molecules to the cytoplasm. As they can easily be linked, nanobodies can serve as “building blocks” to construct multispecific, multivalent, or multiparatopic molecules.

#### Antibody-Based PAI-1 Inhibitors

**Figure 5:**
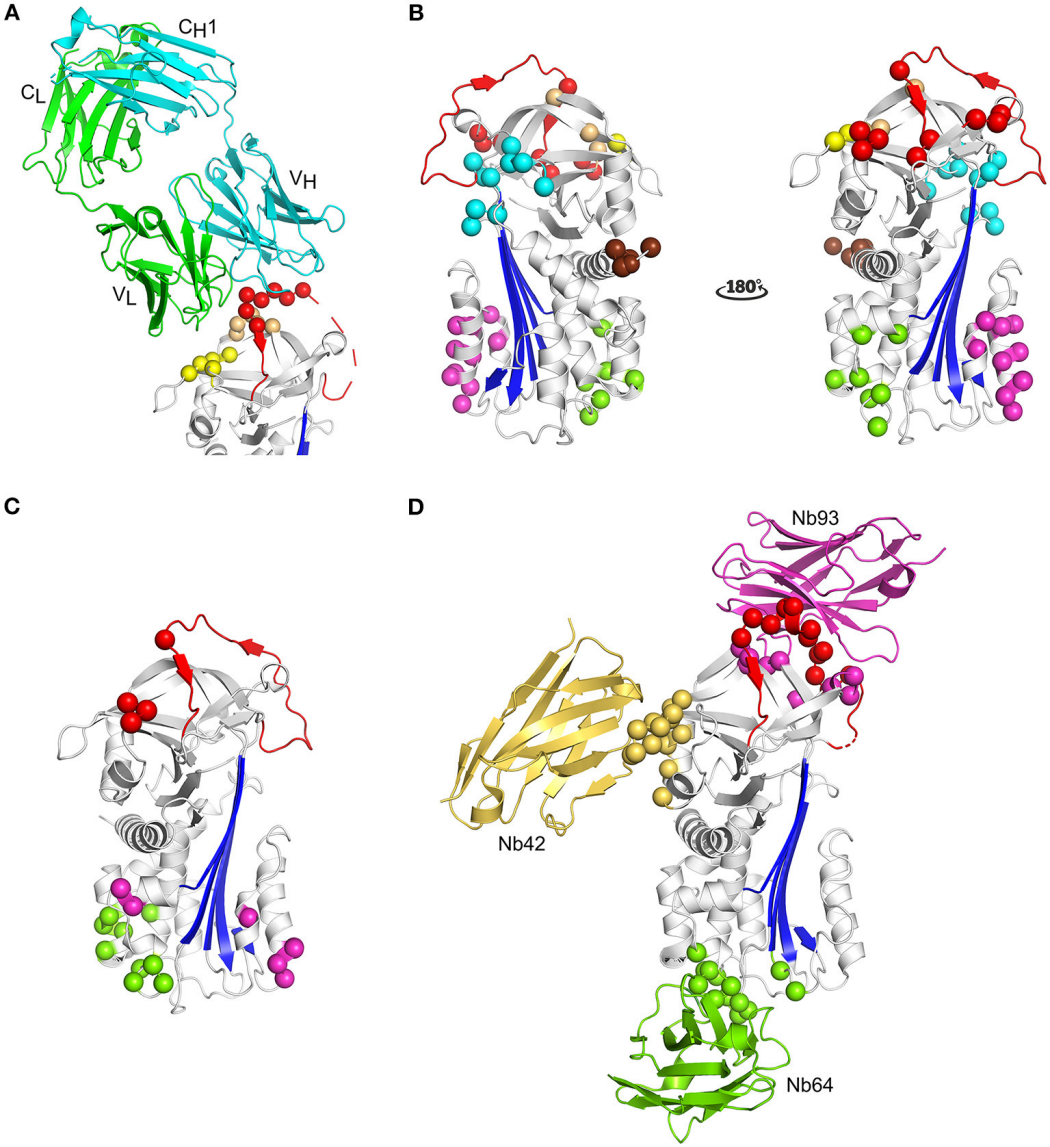
Localization of different epitopes in the structure of active PAI-1. **(A)** Cartoon representation of the crystal structure of PAI-1 in complex with the Fab fragment of MEDI-579 (PDB ID 6I8S) (132). The heavy and the light chain of the Fab fragment are colored cyan and green, respectively. The constant and variable heavy (C_H_1 and V_H_) and light (C_L_ and V_L_) domains are indicated in the figure. Residues closer than 4 Å to MEDI-579 are indicated as spheres. Residues located in the RCL are colored red, residues residing in the exosite regions for the 37-loop and 60-loop of PAs are colored yellow and orange, respectively. **(B)** Localization of different epitopes of monoclonal antibodies (mAbs) as determined by mutagenesis studies. The epitopes of mAbs that prevent the interaction between PAI-1 and PAs are indicated as red (MA-42A2F6, MA-56A7C10, and MA-44E4) (235) and yellow (MA-124K1) (236) spheres. The epitopes of switching mAbs that bind to hF or the hF-s3A loop (MA-33H1F7, MA-55F4C12, and Mab2) (237, 238) are indicated as magenta spheres. The epitope of switching mAb MA-8H9D4 (239) that binds to the hI-s5A loop is indicated as green spheres. The epitopes of latency-inducing antibody MA-33B8 (240, 241), MA-H4B3 (90), and MA-159M12 (242) are indicated as cyan, orange, and brown spheres, respectively. **(C)** Localization of different epitopes of nanobodies as determined by mutagenesis studies (35). The epitope of substrate-inducing nanobody Nb98 is indicated by green and magenta spheres, whereas only the magenta spheres indicate the epitope of Nb64. The epitope of Nb93 that interferes with PAI-1/PA complex formation is indicated as red spheres. **(D)** Cartoon representation of the superimposed crystal structures of PAI-1 in complex with Nb42 [PDB ID 6GWN, 6GWP, and 6GWQ (52)], Nb64 [PDB ID 6GWN and 6GWP (52)], and Nb93 [PDB ID 6ZRV (41)]. Nb42, Nb64, and Nb93 are colored yellow, green, and magenta, respectively. Residues closer than 4 Å to Nb42 are indicated as yellow spheres. Residues closer than 4 Å to Nb64 are indicated as green spheres. Residues closer than 4 Å to Nb93 are indicated as red and magenta spheres. Red spheres represent residues located in the RCL of PAI-1, whereas magenta spheres represent residues located in the exosite binding regions for PAs.

Due to the efforts of many research groups, a large panel of mAbs that interfere with PAI-1 activity is readily available. More recently, nanobody libraries have been constructed as well (35). In contrast to inhibitory peptides and small molecules, the epitopes of antibody-based PAI-1 inhibitors have been mapped to different regions of the PAI-1 molecule (Figure 5). Antibodies have been shown to affect PAI-1 functionality at distinct levels during the PAI-1/PA reaction, i.e., by preventing Michaelis complex formation, by inducing substrate behavior of PAI-1, or by accelerating the conversion to latent PAI-1 (243).

Only recently, the first crystal structure of PAI-1 complexed with an inhibitory antibody fragment belonging to the first class was reported (Table 1) (132). This structure containing the Fab fragment of neutralizing antibody MEDI-579 revealed that its epitope is concentrated around the C-terminal region of the RCL (residues Ala345–Glu350 or P2–P4′) and the neighboring exosites for the 37-loop (Tyr210, Glu212, Tyr220, and Tyr241) and 60-loop (Leu269, Pro270, and Arg271) of PAs (Figure 5A) (132). As a consequence, by simultaneously interfering with exosite interactions and shielding the P1-P1′ reactive center, MEDI-579 prevents the initial interaction between PAI-1 and PAs. A similar mechanism has been described for nanobody VHH-s-a93 (Nb93) that binds an epitope slightly overlapping with that for MEDI-579 (41). The X-ray crystallographic structure of the PAI-1/Nb93 complex revealed that Nb93 binds PAI-1 in a PA-like manner through interactions with the almost full-length RCL and adjacent exosites for PAs other than that for their 37-loop (Table 1 and Figure 5D) (41). In addition, Nb93 was shown to selectively bind and stabilize the active conformation of PAI-1 by anchoring the RCL to the top of the PAI-1 molecule. Notably, similar binding sites including the RCL of PAI-1 and/or neighboring exosites for PAs have been described for other mAbs (Figure 5B), including ESPI-12 (between residues 342–349), MAI-12 (between residues 320–379), MA-42A2F6 (Lys243 and Glu350), MA-56A7C10 (Glu242, Lys243, Glu244, Glu350, Asp355, and Arg356), and MA-44E4 (His185, His186, and Arg187), suggesting that they directly interfere with Michaelis complex formation as well (132, 235, 244, 245). The epitopes of two other nanobodies, VHH-s-a27 (Nb27) and VHH-2g-42 (Nb42), could not be deduced by mutational studies (35). However, it was hypothesized that they might interfere with the PAI-1/PA reaction by directly preventing PAI-1/PA complex formation as well. Indeed, using a hybrid approach employing structural and biochemical methods, Nb42 was shown to destabilize the initial Michaelis complex by binding to the exosite region for the 37-loop of PAs (Table 1 and Figure 5B) (52). Interestingly, MA-124K1 that inhibits rat PAI-1 was found to bind the exosite region for the 37-loop of PAs (Glu212 and Glu220) and thereby inhibits PAI-1 activity while simultaneously enhancing the binding of PAI-1 to vitronectin (236).

The second class of mAbs, referred to as “switching antibodies,” redirect the inhibitory PAI-1/PA reaction toward the substrate branch. Within the category of substrate-inducing antibodies, two different subclasses have been identified. Even though both subclasses ultimately increase the relative fraction of cleaved PAI-1, each class acts through a distinct mechanism by binding different epitope regions localized in the lower half of the PAI-1 molecule (Figure 5B). Several mAbs, including MA-33H1F7 (Glu130, Arg131, and Lys154), MA-55F4C12 (Glu128, Val129, Glu130, Arg131, and Lys154), and Mab2 (Arg131, Arg133, Phe134, Asn137, Asp138, Leu152, and Lys154) were shown to have overlapping epitopes located in hF and the loop connecting hF with s3A of the central PAI-1 β-sheet (237, 238). These mAbs have been shown to slow down the rate of cleaved RCL insertion, possibly by restricting the structural rearrangements within this region during RCL insertion. A different epitope was identified for switching antibody MA-8H9D4 that binds the loop between hI and s5A at the bottom of the PAI-1 molecule (Arg300, Gln303, and Asp305) and possibly residues in hC (Glu53) and hI (Arg287, Glu297, and Asp297) (239). A similar epitope, i.e., comprising residues in hB, hC, and the hI-s5A loop, was identified for nanobody VHH-2w-64 (Nb64). The crystallographic structures of the PAI-1/Nb64 complex later confirmed the crucial involvement of the latter loop (Table 1) (52). Based on structures of other serpin/serine proteinase complexes, the binding site of Nb64 in all probability overlaps with the position of the PA in the final inhibitory complex (Figure 1, Figure 5D). In contrast to the first subclass binding hF, MA-8H9D4 (246), and Nb64 (52) neither affected the formation of the initial PAI-1/PA complex, nor the kinetics of RCL insertion for the PAI-1/tPA reaction. It was therefore suggested that MA-8H9D4 and Nb64 interfere with the final step of inhibitory complex formation by hindering the PA to come close enough to the PAI-1 surface for PA distortion. Furthermore, strong functional additivity has been observed for the MA-33H1F7/MA-8H9D4 and MA-55F4C12/MA-8H9D4 antibody pairs which demonstrates that these mAbs bind structurally distinct epitopes and affect different steps of the PAI-1/PA reaction (246). Importantly, the effects of Mab2, MA-8H9D4, and Nb64 are potentiated by simultaneous binding of vitronectin to the opposite side of hF in PAI-1, which further increases the rigidity within this region (99). Another substrate-inducing nanobody VHH-s-a98 (Nb98) was suggested to bind a cavity aligned by hB and hC (Gln47, Glu53, and Gln55-Gln56-Gln57), the hF-s3A loop (Glu128-Val129-Glu130-Arg131 and Lys154), hI (Glu291 and Asn292), and the hI-s5A loop (Gln303 and Asp305). Since this region harbors binding sites for both subclasses of switching Abs (MA-33H1F7 and MA-55F4C12 vs. MA-8H9D4 and Nb64), the exact mechanism by which Nb98 induces substrate behavior remains unclear.

The third class of mAbs, including MA-35A5, MA-33B8, M5, and MA-H4B3, have the ability to accelerate the active to latent transition of PAI-1 and bind epitopes that are spread more across the PAI-1 surface (Figure 5B). The major determinants of the MA-33B8 epitope were simultaneously reported by two research groups, and are comprised in the same region that covers the turn connecting hD with s2A (Asn87, Lys88, and Asp89), the top of s3A (Gln174 and Lys176), the loop connecting s2B with s3B (His229, Gly230, and Thr232), and the loop connecting s5A with the RCL in the breach region (Asn329 and Ser331) (240, 241). Interestingly, this epitope is relatively less accessible in the active form of PAI-1 and undergoes a structural rearrangement to become more compact in the loop-inserted forms of PAI-1. Furthermore, since the putative epitope contains residues located on both sides of the RCL insertion site, i.e., on s5A and s3A, and MA-33B8 promotes loop insertion, the binding must occur to a prelatent form of PAI-1 in which the RCL is already partially inserted. Additional evidence for a prelatent form that can be accelerated into latency transition has been provided by the binding site and inhibitory mechanisms of M-5 and MA-H4B3. The dominant epitope residue for M-5 was mapped to Asp181 located directly below the RCL at the loop connecting s3A with s4C (247). Since this residue was shown to be more accessible in the latent form, it was hypothesized that M-5 displaces and forces insertion of the RCL into the central β-sheet A upon binding. The epitope of MA-H4B3 includes residues on s1B, s2B, and s3B (Tyr210, Glu212, and Tyr241) as well as s2C (Arg271) and thus partially overlaps with the epitope for MEDI-579 that prevents Michaelis complex formation (90). However, since the epitope of MA-H4B3 was shown to be occluded by s1C in active PAI-1, it was suggested that the epitope only becomes available upon s1C detachment during latency transition. Thus, by binding to a prelatent form that exists in equilibrium with active PAI-1, MA-H4B3 accelerates the rate of RCL insertion, resulting in an enhanced latency transition. Another mAb, MA-159M12, binds to the N-terminal part of hA (Pro2, Leu3, Pro4, and Glu5) and accelerates the active to latent transition in rat PAI-1 (242). However, MA-31C9 that targets a similar region in human PAI-1 (His3, Ser6, Tyr7, and His10) has been shown to be non-inhibitory. Furthermore, introduction of the epitope of MA-159M12 in human PAI-1 only caused MA-159M12 to bind PAI-1 with low affinity. This observation emphasized that two mAbs generated toward the same region in different orthologs can display very divergent functional effects, either caused by subtle structural differences between human and rat PAI-1 or by subtle differences in the binding mode of these mAbs (35, 235–238, 248).

Alternatively, single-chain variable fragments have been derived from several of the aforementioned mAbs (249–251). Since crystallization attempts with these scFvs and their full-size mAbs remained unsuccessful thus far, a mutagenesis approach was used to identify epitope (235, 239) and paratope (251, 252) residues involved in the interaction between PAI-1 and the scFvs. Subsequently, this information was used to drive protein-protein docking in order to predict the structures of the respective PAI-1/scFv complexes (253). Notably, due to the *in vitro* and *in vivo* potency and cross-reactivity toward rodent PAI-1, one scFv (scFv-33H1F7) was developed into a bispecific diabody format together with scFv-TCK26D6 that inhibits the antifibrinolytic enzyme thrombin activatable fibrinolysis inhibitor (TAFI) (254). Further *in vivo* evaluation and comparison with the standard thrombolytic therapy (tPA) showed that the simultaneous administration of MA-33H1F7 and MA-TCK26D6 or the use of diabody Db-TCK26D6x33H1F7 holds a great promise in both prophylaxis and treatment of thrombotic disease (255, 256).

## Conclusions

Over the last four decades, the role of PAI-1 in various pathophysiological processes including cardiovascular disease has been extensively studied (257, 258). As the main physiological inhibitor of PAs, PAI-1 exerts an antifibrinolytic activity mainly by attenuating the plasmin-mediated fibrin degradation and thereby contributes to the pathogenesis of thrombotic cardiovascular diseases. Apart from being a regulator of the plasminogen activation system, PAI-1 has a pleiotropic biological function stemming from its ability to interact with ligands, such as the extracellular matrix protein vitronectin and cellular low-density lipoprotein receptors including LRP1. As a consequence, PAI-1 is also involved in the (patho)physiological processes associated with tissue remodeling, cell migration, and inflammation. Even though the precise role of PAI-1 in these diverse pathological processes is not always fully understood, elevated levels of PAI-1 have been shown to be related to the incidence, severity, and prognosis of various diseases. Therefore, significant clinical interest has been tied to PAI-1 as a putative drug target for the treatment of PAI-1-related pathologies. A very diverse collection of PAI-1 inhibitors has already been developed, including peptides, RNA aptamers, small organochemical molecules, antibodies, and antibody fragments. Even though several antagonists have been extensively characterized *in vitro* and *in vivo*, no PAI-1 inhibitors were approved to date for therapeutic use in humans. However, it should be noted that a few PAI-1 antagonists are currently proceeding through clinical trials, underscoring the persistent clinical interest in safe and efficient modulators of PAI-1 activity. The growing number of available structures from PAI-1 in complex with biological ligands and inhibitors may provide access to useful information for guiding the development of the continuously growing segment of PAI-1 antagonists.

## Author Contributions

MS and PJD contributed to the conception and the design of the manuscript. MS did the literature research, wrote the first draft of the review, and generated the figures and tables. PJD reviewed the manuscript and provided the critical feedback. All authors contributed to manuscript revision, read, and approved the submitted version.

## Conflict of Interest

The authors declare that the research was conducted in the absence of any commercial or financial relationships that could be construed as a potential conflict of interest.
